# The *RB1* Story: Characterization and Cloning of the First Tumor Suppressor Gene

**DOI:** 10.3390/genes10110879

**Published:** 2019-11-01

**Authors:** Jesse L. Berry, Ashley Polski, Webster K. Cavenee, Thaddeus P. Dryja, A. Linn Murphree, Brenda L. Gallie

**Affiliations:** 1 USC Roski Eye Institute, Keck School of Medicine of the University of Southern California, Los Angeles, CA 90033, USA; ashley.polski@usc.edu (A.P.); alm4@mac.com (A.L.M.); 2 The Vision Center at Children’s Hospital Los Angeles, Los Angeles, CA 90027, USA; 3 Ludwig Institute for Cancer Research, University of California, San Diego, CA 92093, USA; wcavenee@ucsd.edu; 4 Department of Medicine, UCSD School of Medicine, San Diego, CA 92093, USA; 5 Moores Cancer Center, UCSD School of Medicine, San Diego, CA 92093, USA; 6 Cogan Eye Pathology Laboratory, Massachusetts Eye and Ear Infirmary, Harvard Medical School, Boston, MA 02114, USA; thaddeus_dryja@meei.harvard.edu; 7 Department of Ophthalmology and Vision Sciences, University of Toronto, Toronto, ON M5T 3A9, Canada; brenda@gallie.ca; 8 Department of Ophthalmology and Vision Sciences, The Hospital for Sick Children, Toronto, ON M5T 3A9, Canada; 9 Departments of Molecular Genetics and Medical Biophysics, University of Toronto, Toronto, ON M5T 3A9, Canada

**Keywords:** retinoblastoma, tumor suppressor gene, cancer genetics, gene cloning, genetic testing

## Abstract

The *RB1* gene is the first described human tumor suppressor gene and plays an integral role in the development of retinoblastoma, a pediatric malignancy of the eye. Since its discovery, the stepwise characterization and cloning of *RB1* have laid the foundation for numerous advances in the understanding of tumor suppressor genes, retinoblastoma tumorigenesis, and inheritance. Knowledge of *RB1* led to a paradigm shift in the field of cancer genetics, including widespread acceptance of the concept of tumor suppressor genes, and has provided crucial diagnostic and prognostic information through genetic testing for patients affected by retinoblastoma. This article reviews the long history of *RB1* gene research, characterization, and cloning, and also discusses recent advances in retinoblastoma genetics that have grown out of this foundational work.

## 1. Introduction

While it is often said in medical school that half of what you learn will be outdated and disproven by the end of medical school, a true paradigm shift in medicine is rare. One such shift has been the understanding of the tumor suppressor gene in cancer genetics and the role these gatekeeper genes play in many human cancers. The first-described tumor suppressor gene initiates retinoblastoma, a pediatric ocular malignancy once generally thought to be the only genetic cancer. Since the discovery and cloning of this gene decades ago, numerous studies of retinoblastoma and related non-ocular tumors have elucidated the molecular and genetic role of *RB1* in cancer development and inheritance. Herein we review the concepts, cloning, characterization and application of the *RB1* retinoblastoma tumor suppressor gene.

## 2. Discussion

### 2.1. Discovery, Characterization, and Cloning of the RB1 Gene

**Figure 1:**
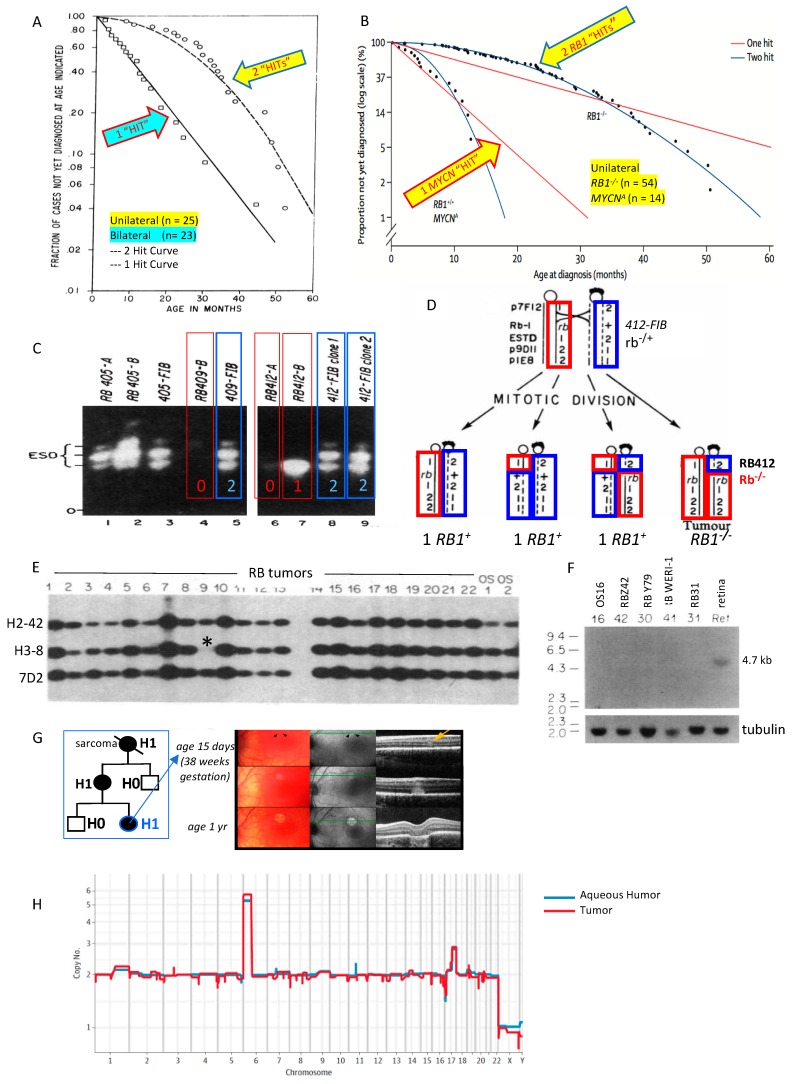
The *RB1* story. (**A**) Knudson evaluated age at diagnosis vs. proportion not diagnosed; two hits initiated unilateral retinoblastoma and only one hit was required for bilateral patients predisposed to retinoblastoma [1]. (**B**) A rare third form of unilateral, non-hereditary retinoblastoma has normal *RB1* alleles and instead is driven by one hit: high-level amplification of the oncogene *MYCN* (reproduced from [23], with permission). (**C**) Inactivation in tumor of esterase D alleles near the retinoblastoma susceptibility locus suggested regional somatic inactivation was the second hit in some retinoblastoma tumors [10]. (**D**) Mitotic recombination results in loss of heterozygosity around *RB1* locus so that cells that form the retinoblastoma tumor have lost both copies of normal *RB1* [11,12]. (**E**) Retinoblastoma tumor 9 (of 22) has homozygous deletion of H-38 probe (*) that mapped in the 13q14 genomic region [16]. (**F**) The *RB1* tumor suppressor gene is cloned: a conserved DNA sequence near H-38 probe hybridized to a 4.7 kb messenger RNA found in all normal tissues but not in retinoblastoma or osteosarcoma tumors [17]. (**G**) Persons carrying a *RB1* pathogenic variant are H1 (at risk for retinoblastoma and second cancers); prenatal detection and early term delivery facilitates detection and laser treatment of invisible tumors with minimal scarring (reproduced from [60], with permission). (**H**) Cell-free DNA from fluid behind the cornea (aqueous humor) shows the same pattern of copy number changes characteristic of retinoblastoma as the tumor from the removed eye (reproduced from [62], with permission).

Historically, retinoblastoma was considered a unique cancer with autosomal dominant inheritance. One of the earliest indications that retinoblastoma is caused by two pathogenic variants, instead of a single activating variant as in an oncogene, was described in 1971 in Knudson’s seminal paper based on the observations of 48 cases of retinoblastoma and associated reports [1]. By evaluating the rates, age, and timing of tumor onset, Knudson concluded that two separate “hits” were required for oncogenesis in retinoblastoma (Figure 1A). The concept of Knudson’s two-hit hypothesis was advanced when Comings proposed that the two hits corresponded to the loss of both alleles of the retinoblastoma cancer susceptibility gene [2]. Soon after, it was recognized that some patients with retinoblastoma have a deletion on the long arm of chromosome 13 [3,4,5,6]. 

Gallie et al. (1978) showed linkage of retinoblastoma in a family supporting the 13q location of the causative gene [5]. Since the human enzyme esterase D was known to be overexpressed in children with trisomy 13, esterase D expression was evaluated in retinoblastoma patients with partial deletions or duplications of chromosome 13 [7]. Murphree and others demonstrated low expression of esterase D in patients with partial 13q deletions and retinoblastoma, concluding that the physical location of the gene that imparted retinoblastoma susceptibility was near the esterase D gene on band 13q14 [8,9].

Godbout et al. (1983) [10] (Figure 1C), furthered understanding of Knudson’s observations by evaluating tumor and normal cells of six patients with retinoblastoma who were heterozygous for esterase D electrophoretic allelic variants. While the normal cells in the children expressed both esterase D alleles, the retinoblastoma tumors of 4/6 children expressed only one allele. They concluded that retinoblastoma tumorigenesis requires somatic inactivation of the retinoblastoma *RB1* locus near the esterase D locus. This was the “first clear evidence of somatic inactivation of a gene linked to a known human cancer-causing gene” [10].

Cavenee et al. (1983) further delineated the recessive nature of the gene predisposing to retinoblastoma by using chromosomal molecular markers to show that tumorigenesis coincides with the loss of heterozygosity around the *RB1* locus resulting from mitotic nondisjunction or recombination [11,12] (Figure 1D). Familial retinoblastoma studies in 1985 and 1986 provided more support to the idea that (1) susceptibility to tumor formation could be inherited and (2) a second somatic pathogenic variant initiates tumor formation, validating the recessive nature of the predisposing genetic variant [13,14]. Hansen et al. (1985) and Dryja et al. (1986) subsequently demonstrated that osteosarcoma—one of the common second primary cancers to affect patients who carry a pathogenic variant in *RB1*—also showed somatic loss of heterozygosity in the region of chromosome 13 that contains the *RB1* locus in patients both with and without retinoblastoma. This bolstered evidence for a recessive mechanism on chromosome 13q14 underlying tumorigenesis in both cancers [15,16]. 

Concurrently, advanced molecular mapping studies refined the chromosomal locus of the susceptibility gene. First, Dryja et al. (1986) identified three cloned DNA fragments from chromosome 13, one of which (H3-8) was missing in 2/37 retinoblastoma tumors (Figure 1E) [16]. H3-8 was then used to obtain neighboring DNA sequences. One of those neighboring sequences was found to be highly conserved across mammals and was therefore chosen to screen a retinal cDNA library created by Friend et al [17]. A transcribed message from the region was identified and cloned as cDNA (clone p4.7R). The transcript was expressed in all tested tissues, but not in retinoblastomas (Figure 1F). Others confirmed this site as the *RB1* gene with further cloning and sequencing [18,19,20]. The esterase D gene was subsequently cloned and shown to be normal in most retinoblastoma tumors, demonstrating that it was separate from the *RB1* locus [21]. 

In 1986, it was also shown that retinoblastoma tumor formation was not necessarily dependent on the oncogene *MYCN*, which was commonly amplified and highly expressed in retinoblastomas (as in neuroblastoma) that had lost *RB1*, likely contributing to tumor progression but not initiation [22]. This was important because a large cohort of researchers felt at that time that dominantly activated genes such as *MYCN*, instead of recessive suppressor genes, were the primary mechanism for malignant tumor formation (see below). Interestingly, in 2013, it was recognized that a rare, non-heritable, aggressive form of retinoblastoma is actually driven by high-level *MYCN* amplification with normal *RB1* genes [23] (Figure 1B). 

Additional studies provided structural evidence for the *RB1* gene as the prototypic recessive human cancer gene, supporting Knudson’s hypothesis [19]. The entire genomic region of the *RB1* locus was cloned, all of its exons and introns were mapped, and numerous high-frequency polymorphisms within the locus were identified. This allowed for very precise genetic testing for the majority of families with the disease (see below) [24]. The refined characterization of the *RB1* gene and its numerous intragenic polymorphisms facilitated the identification of oncogenic point variants in tumors that had no deletions of the gene [20], the discovery that most new germline mutations were derived from sperm rather than ova [25,26], and the initial characterization of the protein from the *RB1* locus (called at that time the antioncogene) [27]. Finally, in 1993, the 180 kb genomic region encoding the *RB1* transcript was sequenced in its entirety; at the time, this was the longest stretch of sequenced human DNA [28]. After the entire human genome sequence was completed, it became apparent that genes *RB1* and esterase D are separated by approximately 1.5 million bp, which confirmed the previously predicted distinction between the esterase D and *RB1* loci [29]. 

### 2.2. Retinoblastoma Genetics Since the Cloning of RB1

Retinoblastoma and the *RB1* tumor suppressor gene led to a paradigm shift in cancer genetics in the late 1980s and early 1990s, with increased acceptance and understanding of the concept and molecular function of tumor suppressor genes [30]. This paved the way for further research to discover other human tumor suppressor genes, the mechanisms of tumorigenesis in human cancers and, particularly, the role of the retinoblastoma protein (pRB) in retinoblastoma and other cancers [31]. 

Since the cloning of *RB1*, the biological function of the *RB1* gene and its protein product (pRB) has become well-known in molecular genetics and cancer research. In normal cells, the hypophosphorylated form of pRB regulates progression through the cell cycle by interacting with the cellular E2F transcription factor to ultimately block transition from the G1 phase to the S phase [32,33,34]. By imposing a suppressive effect on the cell cycle, pRB thus provides a protection against otherwise uncontrolled cell cycling and proliferation. When both alleles of the *RB1* gene are lost—as in the setting of retinoblastoma—the function of pRB is curtailed, resulting in abnormal cell proliferation and tumor formation. Multiple studies have since associated the loss of function of pRB not only with retinoblastoma but with multiple other non-ocular malignancies, including osteosarcoma, cutaneous melanoma, and soft tissue sarcomas [35,36].

Improved technologies in molecular genetics have also expanded our understanding of the specific pathogenic variants that disrupt the function of *RB1*. The 180 kb *RB1* gene has 27 coding exons and a core promoter. The majority (85%) of *RB1* pathogenic variants in retinoblastomas are within this locus [37]. A variety of null pathogenic variants (missense, nonsense, and splicing mutations, small deletions and insertions (indels), as well as promoter mutations) have been found in retinoblastomas, osteosarcomas, and some other tumor types [20,38,39]. Aberrant hypermethylation of the promoter region was found to be a novel mechanism that, in some tumors, inactivated the gene without changing its DNA sequence [40]. Pathogenic intronic variants were found that led to missplicing or a weakly functional protein product [41]. The pathways affected and interaction with the E2F family of transcription factors [42,43,44,45], and the role of the *RB1* protein product in cell cycle regulation and genomic stability [46,47,48,49], are the bases for elucidating the molecular etiology of the cellular pathways underlying retinoblastoma tumorigenesis.

The emergence in embryogenesis of a new pathogenic *RB1* variant in nonfamilial retinoblastoma may be early, with 100% of cells affected, or late, resulting in somatic mosaicism and less than 1% of cells carrying the pathogenic variant [50,51]. As technology facilitates detection of *RB1* pathogenic variants and mosaicism [52], it is important that clinical labs report the frequency of *RB1* pathogenic variants found among bilaterally affected retinoblastoma patients (the *RB1* sensitivity of the test) to support accurate, informed genetic counseling for patients with retinoblastoma and their families. 

As a result of this expanded understanding of *RB1* and its role in heritable and non-heritable retinoblastoma, a new component (“H”) was incorporated into the 8th edition (2017) American Joint Committee on Cancer (AJCC) Cancer Staging Manual for retinoblastoma [53]. By this staging system, retinoblastoma patients may be placed in one of three genetic categories based on peripheral blood analysis: (1) those carrying a cancer-predisposing *RB1* pathogenic variant (H1), (2) those with normal *RB1* alleles (H0), and (3) those with an unknown or unexamined *RB1* status (HX). A fourth category, H0*, has also been proposed to describe patients with <1% residual risk of a *RB1* pathogenic variant due to undetectable mosaicism [54]. 

Genetic testing for *RB1* pathogenic variants in the blood of retinoblastoma patients is now a standard component of the retinoblastoma evaluation. Especially in cases with no prior family history of the disease, genetic analysis can confirm the diagnosis of H0* retinoblastoma and allow a more informed approach for the overall management and counseling of these patients and their families. While all patients with bilateral retinoblastoma can be presumed to have a germline *RB1* pathogenic variant, this is particularly critical for patients with unilateral disease and no prior family history of retinoblastoma, since >15% of such isolated cases harbor a germline (or mosaic) pathogenic variant which alters the prognosis for future ocular tumors, secondary non-ocular tumors, and the risk of passing the trait to future offspring (lack of familial history and older age does not exclude a germline *RB1* gene mutation) [55,56]. There are multiple benefits of genetic testing in retinoblastoma, including a reduction in clinical costs when early diagnosis enables less invasive therapies for H1 persons [57], improved risk assessment for patients and family members, and prevention of unnecessary screening and treatment for H0 relatives [58]. Prenatal knowledge that an infant is H1 opens the potential for early term delivery and detection of nascent invisible tumors by optical coherence tomography for precision laser therapy (Figure 1G) [59,60]. 

Additional required mechanisms of tumorigenesis beyond *RB1* loss have been described [61], although the stepwise progression and exact mechanisms of subsequent genomic changes after *RB1* loss are still not well understood. Retinoblastoma tumor DNA has, up to now, only been available for genomic analysis after the eye was removed. Now, the tumor genome is accessible through cell-free DNA in the anterior segment of the eye, shown to be representative of the tumor, and the pattern of copy number gains characteristic of retinoblastoma may predict which eyes are likely to be salvaged (Figure 1H) [62,63]. 

## 3. Conclusions

Our understanding of retinoblastoma tumorigenesis and heritability has dramatically broadened as a result of the study and cloning of the *RB1* gene. In 2018, the impact of this discovery was recognized when four researchers were honored by the Helen Keller Foundation: A. Linn Murphree, MD; Webster K. Cavenee, PhD; Thaddeus P. Dryja, MD; and Brenda L. Gallie, MD. Application of this understanding of retinoblastoma genetics now supports targeted and informed management in the clinical setting through genetic testing of patients and families. The molecular knowledge of retinoblastoma has stimulated approaches that improve outcomes for affected children and families. There is still much to learn about the complex genetic and molecular etiologies that underlie retinoblastoma development, and the role RB1 plays in the formation of other malignant tumors.
